# A Five-Year Analysis of Market Share and Sales Growth for Original Drugs after Patent Expiration in Korea

**DOI:** 10.1007/s43441-025-00741-x

**Published:** 2025-01-10

**Authors:** Minyoung Bae, Sung Ryul Shim, Dong-Wook Yang, Kyung-Bok Son, Sang-Won Lee

**Affiliations:** 1 School of Pharmacy, Sungkyunkwan University, 2066, Seobu-ro, Jangan-gu, Suwon-si, Gyeonggi-do Republic of Korea; 2 Department of Biomedical Informatics, College of Medicine, Konyang University, Daejeon-si, Republic of Korea; 3 College of Pharmacy, Hanyang University, Ansan-si, Gyeonggi-do, Republic of Korea

**Keywords:** Off-patent original drug, Market share, Sales growth, Anatomical therapeutic chemical classification, Generic competition, Therapeutic market size

## Abstract

**Introduction:**

The sales patterns of original drugs after patent expiration in Korea show a relatively high market share and continuous sales growth differently from those in the U.S. and European countries. This study aims to investigates a five-year sales pattern of original drugs after patent expiration in Korea using empirical data.

**Methods:**

Using data from the Ministry of Food and Drug Safety, original drugs whose patents expired in 2012–2018 were extracted. And we used IQVIA data to determine the market share and sales growth rate of 48 original drugs, whose generic drug launched for the first time in the same molecule market, and whose sales data over five years after first generic entry were available. We analyzed the differences by the attribute of variables.

**Results:**

The sales volume of original drugs in the fifth year (Q 20) had an average growth rate of 150.6% compared with that before the first generic drug launched, indicating a continuous growth. The average market share of original drugs in the fifth year (Q 20) decreased to 70.6%, but it was higher than previously reported research results in Korea and other countries. Differences were observed across the category of attribute.

**Conclusion:**

This study demonstrated that while market share of original drugs is decreasing, the sales volume increased continuously until the fifth year, differently from those of other countries. Variations in sales patterns by attributes reflect unique dynamics in Korea.

**Supplementary Information:**

The online version contains supplementary material available at 10.1007/s43441-025-00741-x.

## Introduction

In the pharmaceutical market, the launch of generic drugs typically causes a sharp decline in the market share and usage of off-patent original drugs. Generic drugs, priced significantly lower, quickly capture market share through aggressive price competition, gradually disappearing original drugs [1–6].

However, Korea displays a unique sales pattern distinct from that of the United States and Europe. Under Korea’s universal health insurance system, pricing and reimbursement policies are critical in shaping market dynamics. When generic drugs enter the market, the original drug’s price is reduced and, after one year, aligns with the price of generic drugs [7]. This pricing structure minimizes active price competition, allowing the market share of off-patent original drugs to remain relatively high or grow in sales volume over time [8–13]. Thus, multinational pharmaceutical companies that enter the Korean market continue to sell even if the patent expires [8, 10].

Despite these unique market characteristics, few studies in Korea have analyzed long-term sales patterns of off-patent original drugs. The U.S. and European research have focused on sales trends of original drugs after patent expiration [3, 4, 5, 6, 14–15] and the impact of generic drug market entry [1, 2, 16–20]. However, Korean studies often examine single-year analyses, ingredient groups, or the timing of generic entry rather than long-term sales trends [21–23]. Research in Korea has primarily addressed regulatory impacts, pricing policies, and generic drug dynamics [8–13, 24–32].

Market exclusivity for new drugs is ensured through patents, which grant exclusive rights to inventions, and data exclusivity, which protects clinical trial data for a defined period. These mechanisms collectively prevent generic entry and incentivize pharmaceutical innovation [33]. Similarly, in Korea, market exclusivity is guaranteed by patents, with data exclusivity uniquely implemented through the Post Market Surveillance (PMS) system, providing a six-year exclusivity period for new drugs [34–35]. Before 2015, however, the marketing approval process for generic drugs was not linked to the patent status of original drugs. As a result, generic drugs often entered the market immediately after the PMS period ended, even if the original drug’s patent was still in force. With the introduction of the patent-approval linkage system in March 2015 [36], generic drugs can now be allowed to enter the market only after the patents listed in the patent list of the Ministry of Food and Drug Safety (MFDS) [37] have expired and PMS period ended or if the generic manufacturer successfully challenges the patents.

In Korea, under general circumstances, when the first generic drug is approved and then listed on the reimbursement formulary of Health Insurance Review and Assessment Service (HIRA) [38], the original drug’s price is reduced to 70% of its initial price. After one year, it is further adjusted to 53.55%. When first listed, generic drugs are priced at 59.5% of the original drug’s initial price and, after one year, are also adjusted to 53.55%, aligning with the original drug’s price [7]. As a result, there is no price difference between original and generic drugs. For highly attractive markets, it is common to see the entry of dozens of generic alternatives; however, active price competition remains rare [25–26].

Despite these distinctive market dynamics, studies that have analyzed sales patterns of original drugs according to the continuous period after patent expiration with empirical sales data remain insufficient in Korea. Thus, this study aims to analyze sales patterns, including the market share and growth rate of original drugs until the fifth year since the first generic drug was launched. This analysis will address key questions such as: What about the sales trend of the original drug? Will the volume growth continue while the market share is decreasing? Do they have different patterns depending on product attributes, generic competition, and market attractiveness?

## Materials and Methods

### Data Collecting

Drug list and data were extracted for original drugs whose patents expired from 2012 to 2018 in the K-Orange Book of MFDS [37]. Individual drugs were classified into 256 products by grouping based on product name, patent registration holder, active ingredient, sales company, and administration route. We excluded 11 products without data from IQVIA data [39] for the entire research period. The drugs were classified into 130 products without generic drugs and 115 products with one or more generic drugs as of December 2023 and then we excluded 8 products whose generic drugs were approved earlier than original drugs to compare the product attribute, generic competition, market attractiveness. Afterwards, among 107 products with generic drugs we excluded 37 drugs whose sales data were not available from the one year before to five years after in IQVIA data [39]. Among 70 products, after we excluded 22 products whose generic drugs of the different therapeutical area in the same molecule were already launched, finally, as a study drug, we selected 48 original drugs.

As Korean regulatory authorities interpret the price listing of the first generic drug as the practical patent expiry and reduce the price of the original drug [7], hence, to analyze the sales patterns of original drugs after the launch of generic drug, for 48 original drugs we measured the listing timing of the first generic drug on the reimbursement formulary of HIRA [38] and defined this as practical patent expiration, thus we searched the sales data for the original drug and market based on this from IQVIA data. [See Supplement 1 Flow chart for selecting study drugs]

### Definition of Original Drug

An original drug, also known as a brand name drug, is a drug approved as a new drug with exclusive market rights. Once its patent expires, generic drugs with the same ingredients, content, and formulation can be launched, offering equivalent safety and efficacy [40–42]. In this study, an original drug refers to one registered through a New Drug Application (NDA) or non-NDA, possessing a unique patent and exclusive rights. A generic drug is defined as having the same ingredients, content, and administration route as the original drug but manufactured by a different company.

## Variables

### Market Share and Growth Rate

This study examines the sales patterns of original drugs up to the fifth-year quarter (Q20) after the launch of the first generic drug. The variables include the market share of the original drug in the same molecule market based on sales value and the sales growth rate compared to one year before generic entry (Q-4). Data for 48 original drugs, including market sales value and product sales value and volume, were collected from IQVIA data (2009–2023) [39]. The analysis by attributes focused on product volume growth using standard unit data to minimize the impact of price changes.

### Product Attribute

The European Pharmaceutical Market Research Association (EphMRA) Anatomical Therapeutic Chemical (ATC) classification [43] was applied at Levels 1, as used by IQVIA.

Formulation information was collected from the Korea MFDS integrated drug information system [37]. The EphMRA New Formulation Code (NFC) [44] classified administration routes into oral drugs (tablet, capsule, granule, powder), injections (vial, ampule, syringe, prefilled syringe, pen), and topicals (cream, ointment, patch, spray).

### Generic Competition

For groups with or without generic drugs, market exclusivity was measured as the period (months) between the original drug’s approval and patent expiry. The period (months) of exclusivity was also calculated from the original drug’s approval to the first generic approval. The period (months) between the patent expiry of the original drug and the approval and launch of the generic drug was also measured. Generic drug counts were measured by identifying drugs with the same active ingredient, content, and administration route up to five years after the first generic’s launch. Relevant data referred to the product information search of MFDS [37] and the drug reimbursement formulary of HIRA [38].

### Market Attractiveness

Market attractiveness was determined by annual product sales and overall market size. Sales value for the original drug and its market (including drugs with similar pharmacological mechanisms at ATC Level 4) were sourced from IQVIA data [39]. For groups without generics, sales in the year before patent expiry were used; for groups with generics, sales in the year before generic entry were applied [See Supplement 2 Variables and data sources].

### Measurement and Statistical Methods

Original drugs were grouped based on the presence of generics. The composition ratio, mean, median, and standard deviation were calculated to compare attributes. Market share and growth rates for 48 original drugs were measured until the five years after generic entry, and the significance of differences in trends (Q − 4 to Q20) for each attribute category was tested using linear regression.

## Results

### Attributes of Off-patent Original Drugs

As shown in Table 1, among ATC codes, the group without generics had the most J, while the group with generics had the most L. For administration routes, oral drugs accounted for the highest proportion in the group with generics (71.0%), while injections were higher in the group without generics (43.1%). The average time to patent expiry was similar between the two groups, at approximately 120 months. In the group with generics, the first generic approval occurred an average of 14.6 months before patent expiry, with market launch 2.4 months later. On average, 24.7 generic drugs were launched per original drug by the fifth year. Sales figures showed significant differences: the average sales for original drugs were KRW 22.6 billion in the group with generics, about 7.8 times higher than KRW 2.9 billion without generics. Similarly, the market size for the group with generics (KRW 116.5 billion) was about 1.9 times larger than those without generics (KRW 60.8 billion).

**Table 1:** Analysis of the attributes of off-patent original drugs

Product attributes	Without generic drug		With generic drug	
	*N* = 130	%	*N* = 107*	%
**ATC code**				
A. Alimentary tract and metabolism	13	10.0	8	7.5
B. Blood and blood forming organs	11	8.5	1	0.9
C. Cardiovascular system	9	6.9	13	12.1
D. Dermatologicals	4	3.1	4	3.7
G. Genito urinary system and sex hormones	11	8.5	9	8.4
H. Systemic hormonal preparations (excluding sex hormones)	6	4.6	2	1.9
J. General anti-infectives systemic	30	23.1	12	11.2
K. Hospital solutions	1	0.8	0	0.0
L. Antineoplastic and immunomodulating agents	7	5.4	19	17.8
M. Musculo-skeletal system	6	4.6	8	7.5
N. Nervous system	15	11.5	17	15.9
P. Parasitology	1	0.8	0	0.0
R. Respiratory system	6	4.6	5	4.7
S. Sensory organs	5	3.8	7	6.5
T. Diagnostic agents	3	2.3	0	0.0
V. Various	2	1.5	2	1.9
**Rout of Administration**				
Oral	58	44.6	76	71.0
Injection	56	43.1	18	16.8
Topical	16	12.3	13	12.1
**Generic competition**				
Mean (Median/S.D.), month	Time from original drug approval to patent expiry^1)^ (*N* = 130)		Time from original drug approval to patent expiry^1)^ and first generic drug approval (*N* = 107*)	
	119.9 (118.0/62.2)		120.5 (118/54.8) and 103.7 (91.0/55.7)	
Mean (Median/S.D.), month			Time from patent expiry^1)^ to first generic drug approval (*N* = 107*)	
			−14.6 (− 6.0/56.5)	
Mean (Median/S.D.), month			Time from patent expiry^1)^ to first generic drug launch (*N* = 85†)	
			2.4 (0.0/40.4)	
Mean (Median/S.D.)			Number of generic drugs launched until the fifth year after the launch of the first generic drug (*N* = 70‡)	
			24.7 (12.0/30.0)	
**Market attractiveness**	**One year before patent expiry**^1)^, **value** (***N*** = **130)**		**One year before the first generic drug launch**,** value** (***N*** = **85†)**	
Mean (Median/S.D.), Local currency, billion KRW	Original drug’s sales		Original drug’s sales	
	2.9 (1.3/5.0)		22.6 (13.0/28.6)	
Mean (Median/S.D.), Local currency, billion KRW	Therapeutic market size (ATC Level 4)		Therapeutic market size (ATC Level 4)	
	60.8 (27.0/77.2)		116.5 (77.9/111.0)	

1) Patent expiry: the timing that protection via patents of the K-Orange Book of MFDS has expired

* 107 of the 115 original drugs with generic drugs, excluding 8 drugs whose generic drugs were approved before the original drug was approved

† 85: Products whose first generic drug launched period was within the IQVIA data collecting period (2009–2023)

‡ 70: Products whose fifth year since the first generic drug was launched is within the IQVIA data collecting period (2009–2023)

### Sales Trends from a Year Before the Generic Drug Launched to Five Years After

#### Market Share and Growth Rate of 48 Original Drugs

**Graph 1:**
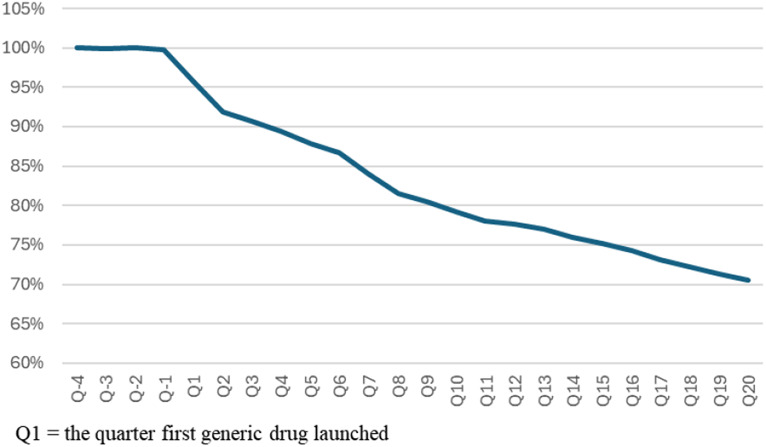
Average market share of 48 original drugs in the same molecule market based on value

**Graph 2:**
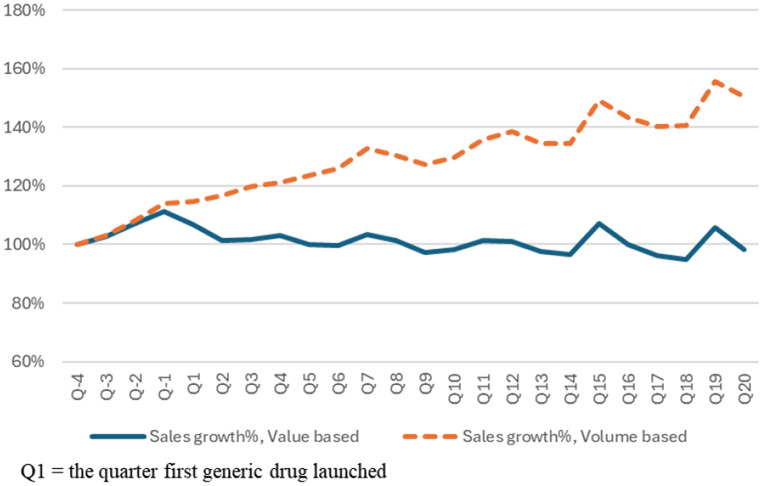
Average sales growth rate, value and volume of the 48 original drugs

The market share of 48 original drugs decreased the most in the first year after the launch of the first generic drug, averaging 89.4% in Q4, 81.5% in Q8, 77.7% in Q12, 74.3% in Q16, and 70.6% in Q20, as depicted in Graph 1. As shown in Graph 2, the sales value growth rate is maintained at an average of 98.3% in Q20 compared to sales of Q − 4, and the sales volume growth rate continued to grow, with an average of 150.6% in Q20, even after generic drugs were launched. [See supplement 3 graphs and tables for more details]

As presented in Table 2, 25.0% of original drugs have a market share of over 90% in Q20. There are 31.3% of original drugs with a value growth rate of more than 100%; 52.1% of original drugs with a volume growth rate of more than 100%; and even 25.0% of products with a volume growth rate of more than 200%.

**Table 2:** Market share and growth rate sub analysis of 48 original drugs

Market share in Q20	Mean (Median/S.D.)		Less than 50%	More than 50% to less than 70%	More than 70% to less than 90%	More than 90%	
	70.6 (75.6/23.5)	N	10	11	15	12	
		%	20.8	22.9	31.3	25.0	
**Growth rate in Q20**	**Mean (Median/S.D.)**		**Less than 50%**	**More than 50% to less than 100%**	**More than 100% to less than 150%**	**More than 150% to less than 200%**	**More than 200%**
Value							
	98.3 (66.9/108.7)	N	18	15	9	2	4
		%	37.5	31.3	18.8	4.2	8.3
Volume							
	150.6 (100.4/133.9)	N	6	17	9	4	12
		%	12.5	35.4	18.8	8.3	25.0

#### Analysis by the Category of Attribute

Table 3 summarizes original drugs’ market share and volume growth rate trends across various attribute categories. The market share and volume growth rate of 48 original drugs were analyzed over five years after the first generic drug launch by the category of product, generic competition, and market attractiveness attributes. The slope of the linear regression line from Q − 4 to Q20 was used to compare sales trends across categories.

**Table 3:** The trend difference analysis of market share and growth rate of 48 original drugs by attributes

Product attributes		Market share (Value)		Sales growth rate (Volume)	
	*N*	Estimate	Pr(>|t|)	Estimate	Pr(>|t|)
**ATC code**					
A. Alimentary tract and metabolism	2	Reference		Reference	
C. Cardiovascular system	9	0.08428	0.016650 *	0.49463	1.42e − 05 ***
G. Genito urinary system and sex hormones	6	−0.04861	0.165522	0.10271	0.358008
H. Systemic hormonal preparations (excluding sex hormones)	2	0.08229	0.019362 *	0.55458	1.29e − 06 ***
J. General anti-infectives systemic	4	0.22802	4.27e − 10 ***	0.4976	1.27e − 05 ***
L. Antineoplastic and immunomodulating agents	10	0.19988	3.30e − 08 ***	0.40333	0.000367 ***
M. Musculo-skeletal system	4	0.06187	0.077928	2.1851	< 2e − 16 ***
N. Nervous system	9	0.10613	0.002662 **	0.42125	0.000202 ***
R. Respiratory system	1	0.12937	0.000267 ***	0.10125	0.364862
V. Various	1	0.08646	0.014074 *	0.16833	0.132546
**Rout of Administration**					
Oral	41	Reference		Reference	
Injection	7	−0.04354	0.241	−0.27301	2.93e − 08 ***
**Generic competition**		**Estimate**	**Pr(>|t|)**	**Estimate**	**Pr(>|t|)**
Number of generic drugs launched in the fifth year after the first generic drug was launched					
More than 1 to less than 10	22	Reference		Reference	
More than 10 to less than 20	8	−0.02799	0.328669	−0.05644	0.56109
More than 20 to less than 30	2	0.03456	0.228196	−0.26316	0.00759 **
More than 30 to less than 40	3	−0.0246	0.390313	0.99621	< 2e − 16 ***
More than 40	13	−0.09858	0.000771 ***	−0.13927	0.15307
**Market attractiveness**		**Estimate**	**Pr(>|t|)**	**Estimate**	**Pr(>|t|)**
Original drug’s sales (One year before the first generic drug was launched, value) Local currency, billion KRW					
Less than 5	8	Reference		Reference	
More than 5 to less than 10	6	−4.87E − 02	0.10659	−1.12894	< 2e − 16 ***
More than 10 to less than 15	5	−3.74E − 02	0.21459	−1.05359	< 2e − 16 ***
More than 15 to less than 20	4	1.13E − 01	0.00025 ***	−0.79099	8.18e − 15 ***
More than 20	25	−1.46E − 05	0.99961	−0.92019	< 2e − 16 ***
Therapeutic market size (ATC Level 4) (One year before the first generic drug was launched, value) Local currency, billion KRW					
Less than 50	19	Reference		Reference	
More than 50 to less than 100	10	−0.03223	0.3	−0.3174	1.34e − 09 ***
More than 100	19	−0.05083	0.104	−0.10132	0.0287 *

The significance of the difference for the linear regression line of the market share and growth rate from Q − 4 to Q20 was tested for each category of individual independent variables. Q1 = the quarter the first generic drug was launched

Significance codes: *** *P* < 0.001 ** *P* < 0.01 * *P* < 0.05

In the analysis by ATC code, original drugs in the J and L groups maintained the highest market share in Q20, while the A and G groups showed the lowest. For administration routes, oral drugs had both a higher market share (72.6%) and greater sales volume growth (159.4%) compared to injections (58.4% and 99.2%, respectively). Injections exhibited a significantly lower growth trend (*p* < 0.001).

**Graph 3:**
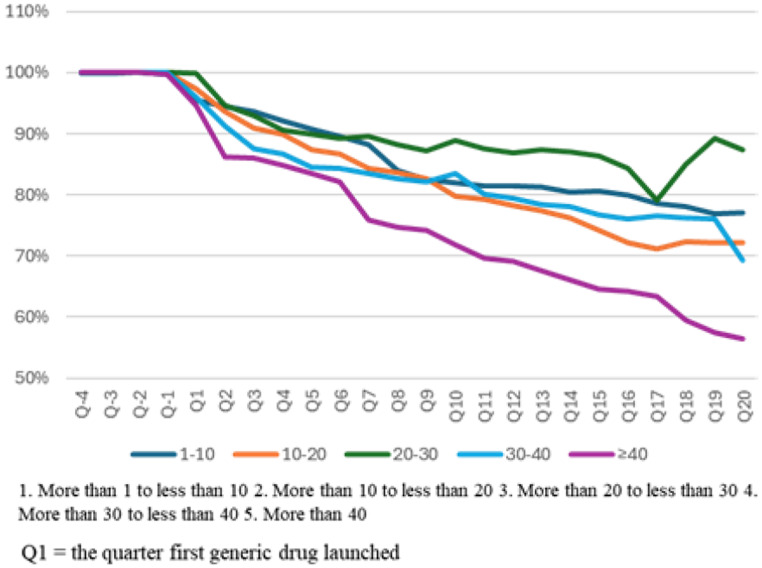
Market share of original drugs in the same molecule market by number of generic drugs launched in five years after the first generic drug launch based on value

When grouped by the number of generic drugs, the market share in Q20 for products with more than 40 generics was significantly lower (56.4%) than for those with fewer generics (*p* < 0.001; Graph 3). Although statistical significance was not observed in all subgroups, steeper market share declines were shown as the number of generic drugs increased. 

For original drug sales, products with higher annual sales tended to show steeper market share declines. Similarly, in the analysis by therapeutic market size, the decline in market share was more pronounced in markets exceeding KRW 100 billion, although these results were not statistically significant. [See supplement 3 graphs and tables for more details]

## Discussion

This study provides three major findings regarding the sales patterns of original drugs and their interaction with generic drugs in the Korean market.

First, generic drugs actively challenge original oral drugs in the Korean market. The time from the original drug’s approval to the first generic approval is shorter compared to studies in Korea (141 months) conducted in 2002 to 2006 [31] and the US (12.9 to 13.6 years) [5–6]. This indicates that generic manufacturers in Korea prepare quickly to enter the market, likely targeting the patent expiration date of original drugs. Additionally, an average of 24.7 generic drugs per original drug was observed, higher than previous studies in Germany (20 generics) [19] and Korea (15.2 generics, conducted in 2012 to 2015) [32]. This trend aligns with global findings, where blockbuster drugs increasingly face intense generic competition [4].

Second, despite declining market share, original drugs maintained sales growth over five years. In this study, market share fell sharply in the first year following generic entry, coinciding with price cuts under the 2012 pricing policy. By the fifth year (Q20), the market share stabilized at 70.6%, notably higher than those in the US and Europe, where generic drugs often dominate the market [1, 5–6], and even higher than the market share (47.0%, 60. 6% respectively) of the Korean studies conducted in 2002 to 2006 [31] and 2012 to 2015 [32]. This suggests that Korea’s pricing structure mitigates the impact of generic entry, allowing original drugs to retain a significant market presence. Furthermore, even after patent expiration, sustained sales growth highlights a unique market dynamic distinct from overseas trends, where original drugs typically phase out rapidly after generic entry.

Third, differences were observed across the categories of product, generic competition, and market attractiveness. Original drugs in the J and L categories retained a high market share over five years, reflecting lower substitution rates. Oral drugs showed more significant market share and growth than injections, which face delayed generic entry due to production challenges [22]. Market share declined more sharply as the number of generics increased, while larger market sizes showed numerically steeper declines, without statistical significance in both cases.

The main reasons why the sales pattern of off-patent original drugs in Korea differs from that of other countries are the drug price system [8, 10–12] and policies [9, 13, 24–26], such as the generic drug prescription activation system, where it is currently not mandatory to prescribe drugs by their generic name in Korea.

First, in Korea since 2012, the price of the original drug become the same as that of generic drugs in the second year [7]. However, the prices of generic drugs are set lower than those of original drugs in European countries, and, generally, generic drugs adopt a cost advantage strategy [19]. However, in Korea, there are cases where the price of the original drug is even lower [28], the cost advantage strategy is rather implemented by the original drug as it has been on the market for many years and can guarantee quality. Studies analyzing the impact of the new drug price policy introduced in Korea in 2012 found that the system was more favorable to original drugs than generic drugs [25] and the impact was temporary, and the policy effect was inefficient and unsustainable due to a lack of demand-side measures [26].

In addition, many countries encourage the use of generic drugs to achieve cost savings by using cheaper generic drugs compared with original drugs [1]. However, the annual savings from using generic drugs in Korea are very low because the price difference between original and generic drugs is small; the price difference among generic drugs is large; and high-priced generic drugs are preferred in the market [28]. Korea also has a policy to encourage alternative preparations, but the replacement rate for low-cost drugs is only 0.2% [9].

In Korea, both off-patent original drugs and generic drugs are growing simultaneously under the current system, so we could not say that the prescription of generic drugs is being encouraged. Therefore, the sales of original drugs continue to grow even after patent expiration, depending on how they are promoted to doctors who prescribe them. Pharmaceutical companies that sell original drugs sometimes adopt differentiation strategies, including ways to improve efficacy, safety, and stability or by developing new substances, new formulations, combination drugs, and new indications [45–47]. In particular, multinational pharmaceutical companies in Korea engage in patent litigation as a defense strategy [45–50], and they actively conduct clinical trials even for off-patent drugs to develop promotional messages.

These findings suggest that Korea’s regulatory and pricing policies allow original drugs to retain significant market share and achieve volume growth despite generic entry. Such dynamics indicate a market environment that prioritizes sustained accessibility to original drugs, which could influence healthcare outcomes and patient adherence positively.

Policymakers and stakeholders should consider how these dynamics affect drug accessibility, competition, and innovation. Further studies are needed to explore the role of promotional strategies, prescribing behaviors, and patient preferences in shaping these trends.

This study has several limitations. First, the impact of drug price changes, patent disputes following the introduction of the patent-approval linkage system [36], and promotional activities for original drugs were not analyzed. Second, factors such as orphan drug designation and the type of active ingredients (e.g., small molecules vs. biopharmaceuticals) may influence market share and exclusivity but were not considered. Biopharmaceuticals follow different pricing schemes, and orphan drugs have longer data protection periods of up to 10 years compared to 4–6 years for other drugs in Korea [51]. These omissions may limit the generalizability of the findings.

Future studies should address these limitations including analyzing competition among original drugs with different active ingredients within the same ATC Level 4 market after patent expiration. Additionally, the role of promotional strategies in influencing sales patterns should be examined, particularly focusing on promotional costs as a share of revenue. Corporate-level factors, such as the proportion of off-patent original drug sales within a company’s portfolio, should also be explored. Finally, further analysis should consider sales patterns based on product characteristics, including formulation types, product types such as orphan drug and active ingredients, as small molecules and biopharmaceuticals may behave differently in the market.

## Conclusion

This study analyzed the sales patterns of 48 original drugs in Korea during the first to fifth years after generic entry, focusing on market share, sales growth, and variations by product attributes, competition, and market attractiveness. While the study drugs do not represent all off-patent original drugs in Korea from 2012 to 2018, it provides key insights into sales patterns immediately following the generic drug launch. Despite decreasing market share, the sales volume of original drugs grew after patent expiration, with market share higher than those reported in previous studies from Korea and other countries. Variations in sales patterns by product attributes, competition, and market attractiveness reflect unique dynamics in Korea’s pharmaceutical market. This study contributes to the literature by examining sales value and volume trends after patent expiration and observing market share erosion and growth patterns of original drugs. These findings emphasize the importance of lifecycle management strategies, such as pricing adjustments, brand loyalty, and effective promotional activities, to maintain the competitive position of off-patent original drugs.

## Electronic Supplementary Material

Below is the link to the electronic supplementary material.

Supplementary Material 1

Supplementary Material 2

Supplementary Material 3

## Data Availability

No datasets were generated or analysed during the current study.
