# Supplementary figures and images for: A Five-Year Analysis of Market Share and Sales Growth for Original Drugs after Patent Expiration in Korea
Source: Ther Innov Regul Sci. 2025 Jan 10;59(2):349–58. doi: 10.1007/s43441-025-00741-x (PMC11880101; doi:10.1007/s43441-025-00741-x)

## Flow chart for selecting study drugs

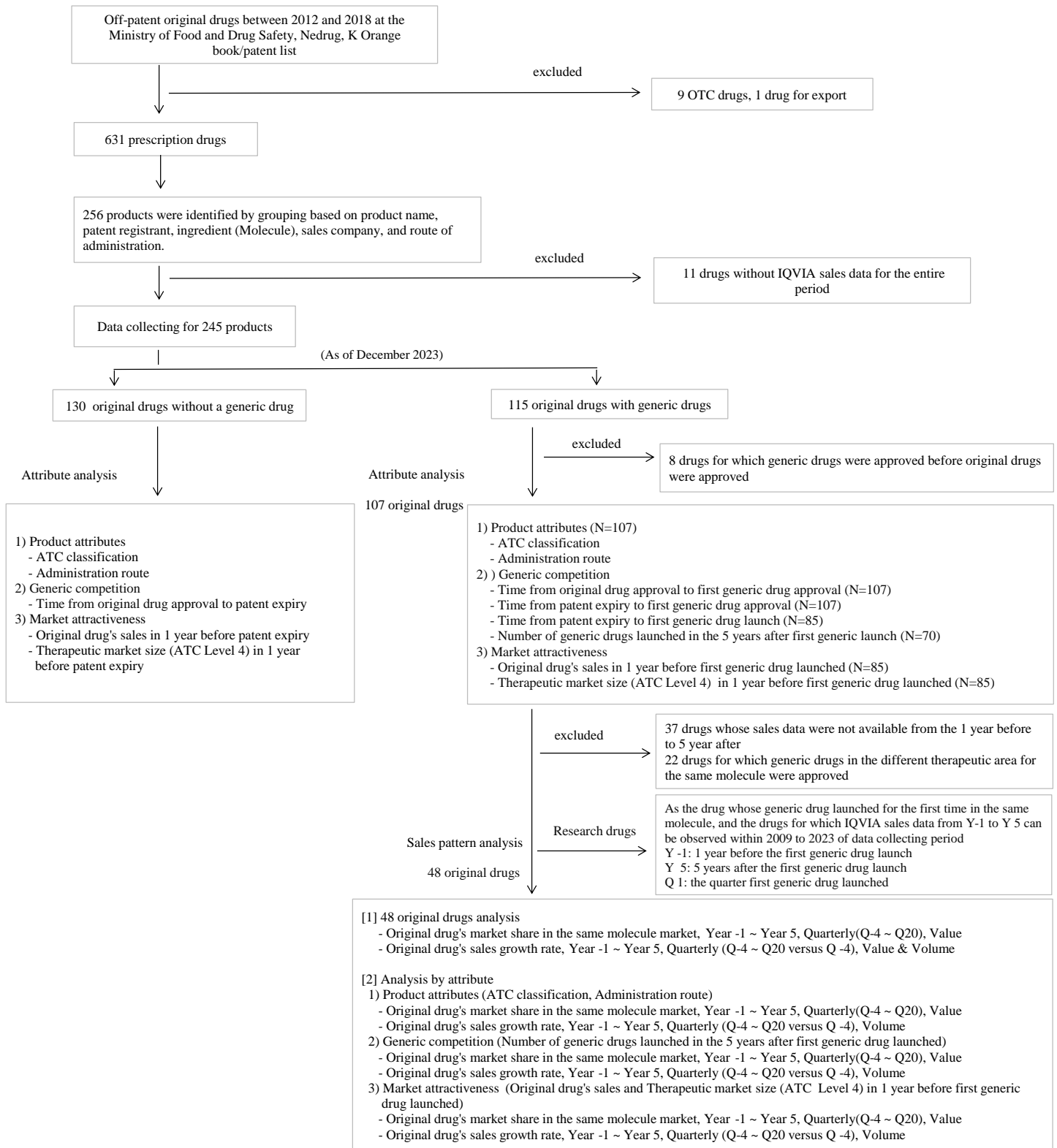

Supplement: Supplementary file 1 — Supplementary Material 1 [file 43441_2025_741_MOESM1_ESM.pdf]
