# Supplementary material for: A Five-Year Analysis of Market Share and Sales Growth for Original Drugs after Patent Expiration in Korea
Source: Ther Innov Regul Sci. 2025 Jan 10;59(2):349–58. doi: 10.1007/s43441-025-00741-x (PMC11880101; doi:10.1007/s43441-025-00741-x)
Supplement: Supplementary file 2 — Supplementary Material 2 [file 43441_2025_741_MOESM2_ESM.pdf]

Variables and data sources

	Variable Items	Data source	Category
**Primary Outcome**	Market share of original drugs after first generic drug launch in the same molecule market, Quarterly (Q -4 to Q 20), Q 1: the quarter first generic drug launched, based on value	IQVIA, National Sales Audit data(Combined)<br>Original drug's sales value, relevant molecule market's sales value	
**Secondary Outcome**	Sales growth rate original drugs after first generic drug launch, Quarterly (Q -4 to Q 20) compared with Q-4, Q 1: the quarter first generic drug launched, based on value and volume	IQVIA, National Sales Audit data(Combined)<br>Original drug's sales value and volume<br>Volume: Standard Unit implemented	
**Product attributes**	ATC (Anatomical Therapeutic Chemical) Classification	*MFDS website: https://nedrug.mfds.go.kr/searchDrug (ATC code)<br>*EphMRA ATC classification: implemented in product and market sales data by IQVIA (ATC level 1), Same Molecule level: implemented by same active ingredient	ATC level 1 A ~ V, 16 Types<br>A. ALIMENTARY TRACT AND METABOLISM<br>B. BLOOD AND BLOOD FORMING ORGANS<br>C. CARDIOVASCULAR SYSTEM<br>D. DERMATOLOGICALS<br>G. GENITO URINARY SYSTEM AND SEX HORMONES<br>H. SYSTEMIC HORMONAL PREPARATIONS (excluding sex hormones)<br>J. GENERAL ANTI INFECTIVES SYSTEMIC<br>K. HOSPITAL SOLUTIONS<br>L. ANTINEOPLASTIC AND IMMUNOMODULATING AGENTS<br>M. MUSCULO SKELETAL SYSTEM<br>N. NERVOUS SYSTEM<br>P. PARASITOLOGY<br>R. RESPIRATORY SYSTEM<br>S. SENSORY ORGANS<br>T. DIAGNOSTIC AGENTS<br>V. VARIOUS
	Route of administration by Formulation	*MFDS website: https://nedrug.mfds.go.kr/searchDrug (formulation information)<br>*IQVIA 2023 NFC(New Formulation Code) Poster	1.Oral 2.Injection 3.Topical<br>IQVIA NFC(New Formulation Code) data<br>1.Oral: Tablet,Capsule,Granule,Powder<br>2.Injection: Vial, Ampule, Syringe, Prefilled syringe, Pen 3.Topical: Cream, Ointment, Patch, Spray
**Generic competition**	Number of generic drugs launched until 5 years after first generic drug launch	*HIRA website: https://www.hira.or.kr/bbsDummy.do?pgmid=HIRAA030014050000<br>*MFDS website: https://nedrug.mfds.go.kr/searchDrug (Drug information search)	1. More than 1 to less than 10 2. More than 10 to less than 20 3. More than 20 to less than 30 4. More than 30 to less than 40 5. More than 40
	Time from patent expiry to first generic drug approval and launch(months)	*MFDS website: https://nedrug.mfds.go.kr/searchDrug (Approval date in Drug information search)<br>*HIRA website: https://www.hira.or.kr/bbsDummy.do?pgmid=HIRAA030014050000	
	With or without generic drug: Original drug approval to patent expiry<br>Time from original drug approval to first generic drug approval(months)	*MFDS website: https://nedrug.mfds.go.kr/searchDrug (Approval date in Drug information search)	
**Market attractiveness**	Without generic drug: Original drug's sales in 1 year before patent expiry,<br>(annual product sales, value, local currency, billion KRW)	IQVIA, National Sales Audit data (Combined)	
	Without generic drug: Therapeutic market size (ATC Level 4) in 1 year before patent expiry,<br>(ATC level 4 annual sales, value, local currency, billion KRW)	IQVIA, National Sales Audit data (Combined)	
	With generic drug: Original drug's sales in 1 year before first generic drug launched (annual product sales, value, local currency, billion KRW)	IQVIA, National Sales Audit data (Combined)	1. Less than 5 billion KRW 2. More than 5 to less than 10 billion KRW 3. More than 10 to less than 15 billion KRW 4. More than 15 to less than 20 billion KRW 5. More than 20 billion KRW
	With generic drug: Therapeutic market size (ATC Level 4) in 1 year before first generic drug launched,<br>(ATC level 4 annual sales, value, local currency, billion KRW)	IQVIA, National Sales Audit data (Combined)	1. Less than 50 billion KRW 2. More than 50 to less than 100 billion KRW 3. More than 100 billion KRW
