# Supplementary material for: A Five-Year Analysis of Market Share and Sales Growth for Original Drugs after Patent Expiration in Korea
Source: Ther Innov Regul Sci. 2025 Jan 10;59(2):349–58. doi: 10.1007/s43441-025-00741-x (PMC11880101; doi:10.1007/s43441-025-00741-x)
Supplement: Supplementary file 3 — Supplementary Material 3 [file 43441_2025_741_MOESM3_ESM.pdf]

Supplement graphs and tables

20240812

1. Overall analysis of 48 original drugs

Graph 1. Average market share of 48 Original drugs in the same molecule market, based on value

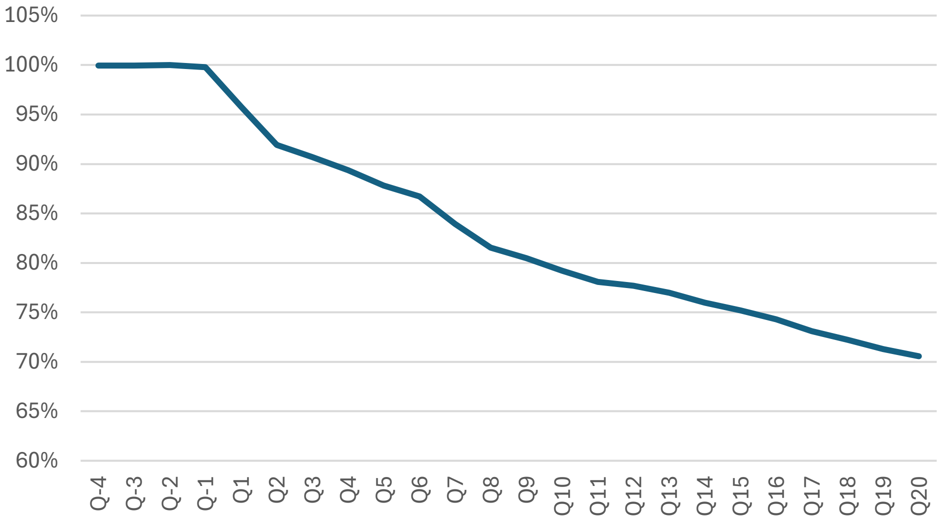

Q1 = the quarter first generic launched

	Year-1				Year 1				Year 2				Year 3				Year 4				Year 5			
	Q-4	Q-3	Q-2	Q-1	Q1	Q2	Q3	Q4	Q5	Q6	Q7	Q8	Q9	Q10	Q11	Q12	Q13	Q14	Q15	Q16	Q17	Q18	Q19	Q20
Mean	100.0%	99.9%	100.0%	99.8%	95.8%	91.9%	90.7%	89.4%	87.8%	86.7%	83.9%	81.5%	80.5%	79.2%	78.1%	77.7%	77.0%	76.0%	75.2%	74.3%	73.1%	72.2%	71.3%	70.6%
Median	100.0%	100.0%	100.0%	100.0%	100.0%	99.5%	97.9%	97.4%	96.3%	94.0%	90.4%	88.3%	87.4%	85.5%	83.4%	82.1%	80.7%	79.0%	80.4%	79.2%	76.5%	76.4%	77.9%	75.6%
S.D.	0.3%	0.4%	0.0%	1.0%	11.5%	15.3%	14.6%	15.8%	17.1%	17.8%	20.4%	21.3%	21.4%	21.7%	22.0%	21.8%	22.7%	22.9%	22.9%	23.2%	23.6%	24.0%	24.0%	23.5%

Graph 2. Average sales growth rate of 48 Original drug, value and volume

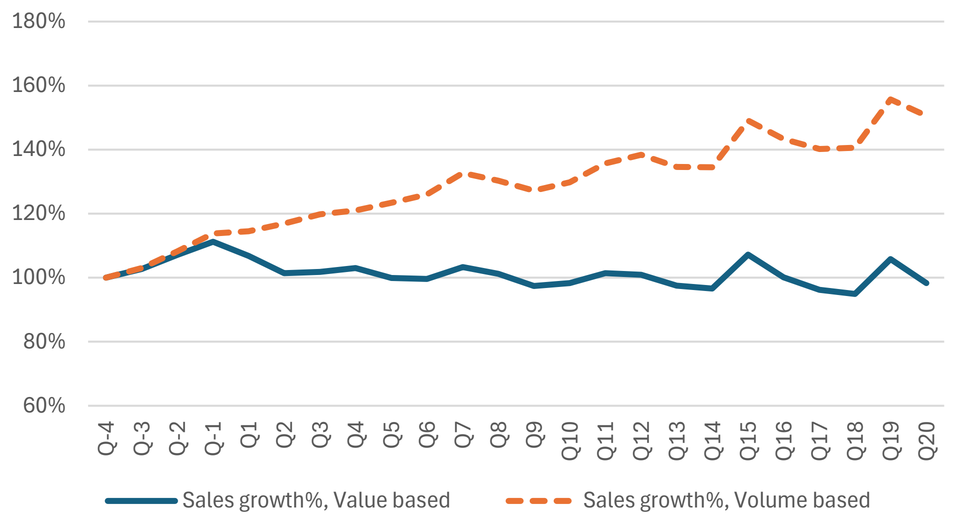

Q1 = the quarter first generic launched

Value based

	Year-1				Year 1				Year 2				Year 3				Year 4				Year 5			
	Q-4	Q-3	Q-2	Q-1	Q1	Q2	Q3	Q4	Q5	Q6	Q7	Q8	Q9	Q10	Q11	Q12	Q13	Q14	Q15	Q16	Q17	Q18	Q19	Q20
Mean	100.0%	102.7%	107.1%	111.2%	106.8%	101.5%	101.8%	103.0%	99.9%	99.6%	103.3%	101.2%	97.4%	98.3%	101.4%	100.9%	97.5%	96.6%	107.3%	100.1%	96.2%	94.9%	105.8%	98.3%
Median	100.0%	101.5%	105.3%	107.3%	105.4%	95.1%	92.6%	92.9%	88.5%	87.0%	83.9%	83.0%	82.1%	77.0%	76.4%	75.0%	72.5%	71.7%	70.1%	69.5%	67.7%	69.2%	71.2%	66.9%
S.D.	0.0%	9.7%	30.2%	41.1%	42.6%	44.6%	61.2%	72.3%	69.6%	74.5%	83.2%	91.1%	90.4%	93.7%	97.3%	105.1%	98.4%	97.0%	123.1%	117.3%	103.7%	98.8%	122.2%	108.7%

Volume based

	Year-1				Year 1				Year 2				Year 3				Year 4				Year 5			
	Q-4	Q-3	Q-2	Q-1	Q1	Q2	Q3	Q4	Q5	Q6	Q7	Q8	Q9	Q10	Q11	Q12	Q13	Q14	Q15	Q16	Q17	Q18	Q19	Q20
Mean	100.0%	103.0%	108.2%	113.8%	114.5%	116.9%	119.8%	121.0%	123.4%	126.0%	132.7%	130.3%	127.2%	129.8%	135.7%	138.4%	134.6%	134.5%	149.1%	143.3%	140.2%	140.6%	155.7%	150.6%
Median	100.0%	102.0%	105.6%	110.0%	107.3%	110.8%	106.0%	107.3%	107.0%	110.3%	108.5%	110.5%	109.5%	103.5%	105.3%	105.8%	101.8%	97.7%	104.8%	105.1%	107.7%	108.6%	104.3%	100.4%
S.D.	0.0%	10.0%	30.1%	39.9%	42.7%	44.4%	61.9%	72.5%	71.1%	76.9%	89.3%	95.5%	97.0%	102.5%	109.7%	126.4%	110.4%	110.8%	136.1%	130.4%	120.6%	119.5%	146.1%	133.9%

2. Analysis by Attributes [Product Charateristics] – ATC

Graph 3. Market share of original drugs in the same molecule market by ATC, based on value

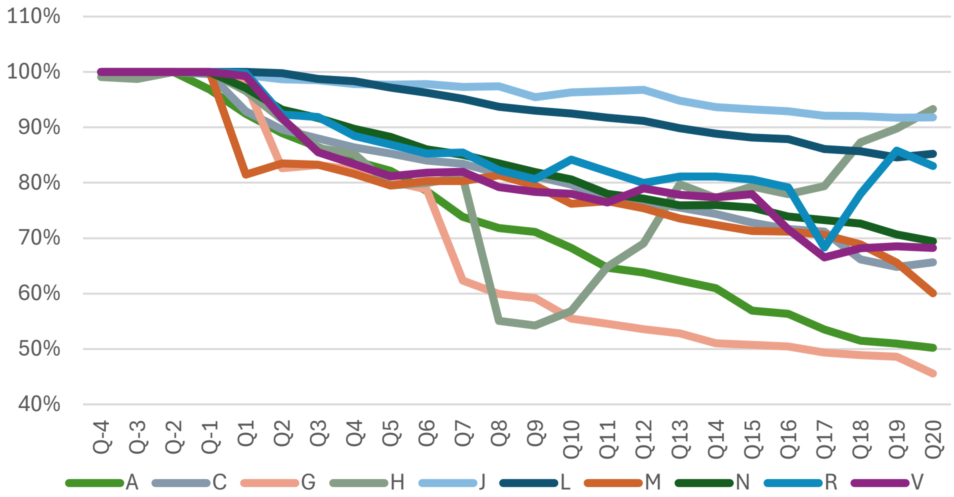

Q1 = the quarter first generic launched

Statistical analysis results

ATC	N		Estimate	Std. Error	t value	Pr(>|t|)
A	2	Reference				
C	9		0.08428	0.03494	2.412	0.016650 *
G	6		-0.04861	0.03494	-1.391	0.165522
H	2		0.08229	0.03494	2.355	0.019362 *
J	4		0.22802	0.03494	6.526	4.27e-10 ***
L	10		0.19988	0.03494	5.720	3.30e-08 ***
M	4		0.06187	0.03494	1.771	0.077928 .
N	9		0.10613	0.03494	3.037	0.002662 **
R	1		0.12937	0.03494	3.702	0.000267 ***
V	1		0.08646	0.03494	2.474	0.014074 *

A. ALIMENTARY TRACT AND METABOLISM B. BLOOD AND BLOOD FORMING ORGANS C. CARDIOVASC ULAR SYSTEM D. DERMATOLOGICALS G. GENITO URINARY SYSTEM AND SEX HORMONES H. SYSTEMIC HORMONAL PREPARATIONS (excluding sex hormones) J. GENERAL ANTI INFECTIVES SYSTEMIC K. HOSPI TAL SOLUTIONS L. ANTINEOPLASTIC AND IMMUNOMODULATING AGENTS M. MUSCULO SKELETAL SYS TEM N. NERVOUS SYSTEM P. PARASITOLOGY R. RESPIRATORY SYSTEM S. SENSORY ORGANS T. DIAGNO STIC AGENTS V. VARIOUS
Signif. codes: '***' 0.001 '**' 0.01 '*' 0.05 '.' 0.1

ATC	N	Q-4	Q-3	Q-2	Q-1	Q1	Q2	Q3	Q4	Q5	Q6	Q7	Q8	Q9	Q10	Q11	Q12	Q13	Q14	Q15	Q16	Q17	Q18	Q19	Q20
A	2	100.0%	100.0%	100.0%	96.8%	92.4%	89.0%	86.4%	84.0%	82.2%	78.5%	73.9%	71.8%	71.1%	68.3%	64.6%	63.8%	62.3%	61.0%	56.9%	56.3%	53.5%	51.5%	51.0%	50.2%
C	9	100.0%	100.0%	100.0%	99.6%	92.9%	89.6%	88.0%	86.4%	85.3%	84.0%	83.4%	81.7%	81.2%	79.7%	76.7%	76.5%	75.5%	74.3%	72.8%	71.7%	71.2%	66.1%	64.8%	65.7%
G	6	100.0%	100.0%	100.0%	100.0%	97.9%	82.6%	83.2%	82.3%	80.2%	78.8%	62.4%	59.9%	59.2%	55.4%	54.5%	53.6%	52.8%	51.0%	50.8%	50.4%	49.4%	48.9%	48.6%	45.6%
H	2	99.1%	98.8%	100.0%	100.0%	96.6%	91.5%	86.2%	85.2%	79.5%	80.0%	81.0%	55.1%	54.2%	56.8%	64.9%	69.1%	79.7%	77.2%	79.3%	77.9%	79.4%	87.3%	89.8%	93.3%
J	4	100.0%	100.0%	100.0%	100.0%	99.4%	98.7%	98.5%	97.8%	97.7%	97.8%	97.3%	97.4%	95.5%	96.3%	96.5%	96.8%	94.8%	93.6%	93.3%	92.9%	92.1%	92.1%	91.7%	91.8%
L	10	100.0%	100.0%	100.0%	100.0%	100.0%	99.8%	98.8%	98.3%	97.2%	96.3%	95.1%	93.7%	93.0%	92.5%	91.7%	91.2%	89.9%	88.9%	88.1%	87.9%	86.1%	85.7%	84.6%	85.2%
M	4	100.0%	100.0%	100.0%	100.0%	81.5%	83.5%	83.2%	81.6%	79.5%	80.3%	80.3%	81.3%	79.5%	76.2%	76.7%	75.4%	73.6%	72.4%	71.3%	71.2%	70.6%	68.9%	65.6%	60.1%
N	9	100.0%	100.0%	100.0%	99.9%	97.1%	93.2%	91.7%	89.7%	88.3%	86.0%	85.0%	83.5%	81.9%	80.6%	78.0%	77.2%	75.9%	76.0%	75.5%	73.9%	73.3%	72.6%	70.7%	69.4%
R	1	100.0%	100.0%	100.0%	100.0%	99.8%	92.3%	91.9%	88.5%	86.9%	85.3%	85.4%	82.3%	80.7%	84.2%	82.1%	80.0%	81.1%	81.1%	80.6%	79.2%	68.3%	78.0%	85.8%	83.0%
V	1	100.0%	100.0%	100.0%	100.0%	99.2%	91.7%	85.5%	83.4%	81.2%	81.8%	82.0%	79.2%	78.4%	78.0%	76.4%	79.0%	77.8%	77.4%	77.9%	71.6%	66.5%	68.2%	68.5%	68.3%

A. ALIMENTARY TRACT AND METABOLISM B. BLOOD AND BLOOD FORMING ORGANS C. CARDIOVASCULAR SYSTEM D. DERMATOLOGICALS G. GENITO URINARY SYSTEM AND SEX HORMONES H. SYSTEMIC HORMONAL PREPARATIONS (excluding sex hormones) J. GENERAL ANTI INFECTIVES SYSTE MIC K. HOSPITAL SOLUTIONS L. ANTINEOPLASTIC AND IMMUNOMODULATING AGENTS M. MUSCULO SKELETAL SYSTEM N. NERVOUS SYSTEM P. PARASITOLOGY R. RESPIRATORY SYSTEM S. SENSORY ORGANS T. DIAGNOSTIC AGENTS V. VARIOUS

2. Analysis by Attributes [Product Charateristics] - ATC

Graph 4. Sales growth rate of Original drugs by ATC, based on Volume

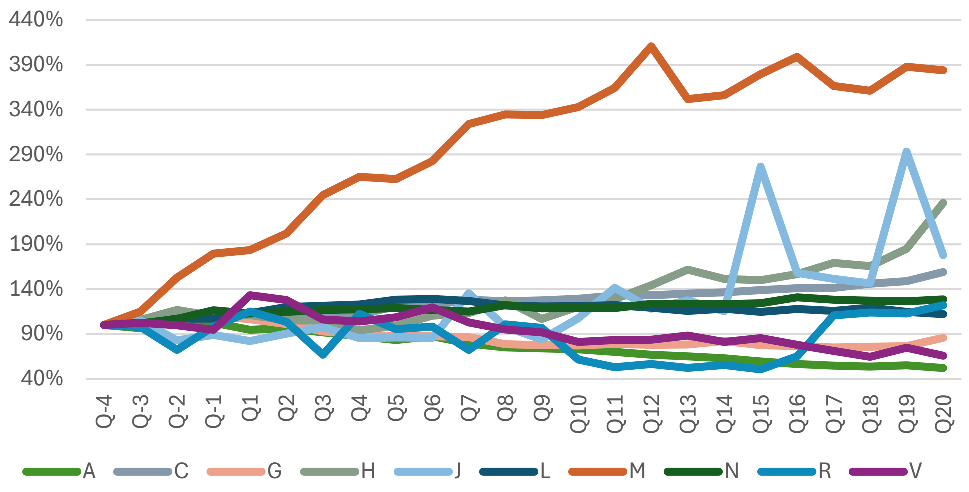

Q1 = the quarter first generic launched

Statistical analysis results

ATC	N		Estimate	Std. Error	t value	Pr(>|t|)
A	2	Reference				
C	9		0.49463	0.11152	4.435	1.42e-05 ***
G	6		0.10271	0.11152	0.921	0.358008
H	2		0.55458	0.11152	4.973	1.29e-06 ***
J	4		0.49760	0.11152	4.462	1.27e-05 ***
L	10		0.40333	0.11152	3.617	0.000367 ***
M	4		2.18510	0.11152	19.594	< 2e-16 ***
N	9		0.42125	0.11152	3.777	0.000202 ***
R	1		0.10125	0.11152	0.908	0.364862
V	1		0.16833	0.11152	1.509	0.132546

A. ALIMENTARY TRACT AND METABOLISM B. BLOOD AND BLOOD FORMING ORGANS C. CARDIOVASCULAR SYSTEM D. DERMATOLOGICALS G. GENITO URINARY SYSTEM AND SEX HORMONES H. SYSTEMIC HORMONAL PREPARATIONS (excluding sex hormones) J. GENERAL ANTI INFECTIVES SYSTEMIC K. HOSPITAL SOLUTIONS L. ANTINEOPLASTIC AND IMMUNOMODULATING AGENTS M. MUSCULO SKELETAL SYSTEM N. NERVOUS SYSTEM P. PARASITOLOGY R. RESPIRATORY SYSTEM S. SENSORY ORGANS T. DIAGNOSTIC AGENTS V. VARIOUS
Signif. codes: '***' 0.001 '**' 0.01 '*' 0.05 '.' 0.1

ATC	N	Q-4	Q-3	Q-2	Q-1	Q1	Q2	Q3	Q4	Q5	Q6	Q7	Q8	Q9	Q10	Q11	Q12	Q13	Q14	Q15	Q16	Q17	Q18	Q19	Q20
A	2	100.0%	96.4%	103.7%	102.3%	94.8%	96.5%	91.7%	87.6%	83.0%	87.4%	79.3%	75.1%	73.7%	72.9%	69.8%	66.7%	65.0%	62.7%	59.4%	56.4%	54.5%	53.5%	54.9%	52.0%
C	9	100.0%	102.6%	108.0%	110.7%	110.1%	107.8%	110.3%	111.6%	120.6%	121.9%	128.1%	126.5%	127.4%	129.1%	132.7%	133.1%	134.7%	136.2%	138.9%	140.9%	141.2%	146.0%	148.9%	158.9%
G	6	100.0%	102.5%	105.6%	110.2%	106.2%	100.8%	92.8%	89.5%	87.6%	87.4%	86.5%	78.6%	77.4%	77.0%	78.8%	77.7%	78.3%	82.0%	77.6%	76.5%	74.9%	75.8%	76.5%	85.7%
H	2	100.0%	105.6%	116.9%	109.8%	110.8%	105.2%	104.5%	94.0%	99.1%	110.3%	113.1%	127.5%	106.6%	120.4%	128.7%	144.0%	161.7%	151.3%	150.0%	156.6%	169.2%	165.7%	184.8%	236.0%
J	4	100.0%	105.0%	82.9%	88.8%	82.0%	90.4%	97.4%	85.3%	85.9%	85.7%	135.2%	97.9%	84.0%	107.4%	141.3%	118.0%	127.5%	115.2%	276.4%	158.0%	151.4%	146.2%	293.2%	177.7%
L	10	100.0%	100.8%	107.0%	106.9%	113.3%	119.9%	121.3%	122.9%	127.9%	129.0%	126.9%	121.6%	120.5%	120.9%	122.2%	119.2%	115.8%	118.0%	114.4%	117.7%	115.6%	119.0%	114.6%	111.9%
M	4	100.0%	115.0%	152.9%	179.4%	183.3%	201.9%	244.8%	265.1%	262.8%	282.6%	324.1%	334.6%	333.9%	342.7%	364.2%	410.6%	351.9%	356.1%	379.7%	398.8%	366.2%	361.1%	387.7%	383.8%
N	9	100.0%	101.6%	106.8%	116.5%	112.8%	114.6%	116.8%	117.3%	118.8%	116.7%	114.9%	122.1%	118.5%	118.8%	118.9%	123.5%	123.4%	123.0%	124.1%	130.8%	128.1%	127.2%	126.4%	128.4%
R	1	100.0%	97.8%	72.1%	98.0%	115.1%	103.4%	66.9%	112.4%	95.2%	98.2%	72.3%	100.7%	96.8%	61.4%	52.7%	56.5%	52.1%	55.5%	50.5%	65.1%	110.8%	113.8%	112.9%	121.9%
V	1	100.0%	102.6%	99.6%	94.7%	133.1%	127.9%	106.1%	103.9%	108.5%	119.7%	102.9%	94.8%	91.8%	81.2%	83.1%	83.5%	88.1%	81.0%	85.2%	77.9%	70.9%	64.5%	74.6%	65.6%

A. ALIMENTARY TRACT AND METABOLISM B. BLOOD AND BLOOD FORMING ORGANS C. CARDIOVASCULAR SYSTEM D. DERMATOLOGICALS G. GENITO URINARY SYSTEM AND SEX HORMONES H. SYSTEMIC HORMONAL PREPARATIONS (excluding sex hormones) J. GENERAL ANTI INFECTIVES SYSTEMIC K. HOSPITAL SOLUTIONS L. ANTINEOPLASTIC AND IMMUNOMODULATING AGENTS M. MUSCULO SKELETAL SYSTEM N. NERVOUS SYSTEM P. PARASITOLOGY R. RESPIRATORY SYSTEM S. SENSORY ORGANS T. DIAGNOSTIC AGENTS V. VARIOUS

2. Analysis by Attributes [Product Charateristics] - Route of administration

Graph 5. Market share of original drugs in the same molecule market by formulation, based on value

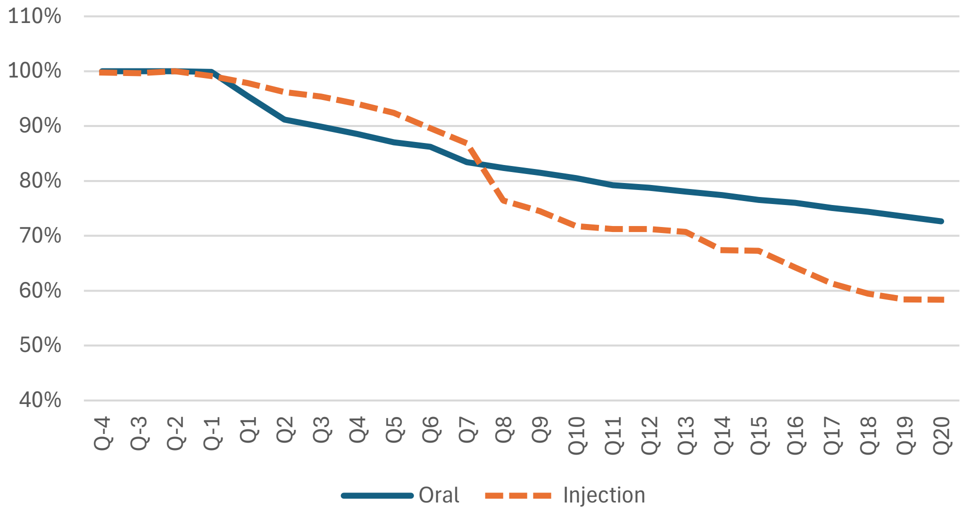

Q1 = the quarter first generic launched

Statistical analysis results

Formulation	N		Estimate	Std. Error	t value	Pr(>|t|)
Oral	41	Reference				
Injection	7		-0.04354	0.03668	-1.187	0.241

Signif. codes: '***' 0.001 '**' 0.01 '*' 0.05 '.' 0.1

	N	Q-4	Q-3	Q-2	Q-1	Q1	Q2	Q3	Q4	Q5	Q6	Q7	Q8	Q9	Q10	Q11	Q12	Q13	Q14	Q15	Q16	Q17	Q18	Q19	Q20
Oral	41	100.0%	100.0%	100.0%	99.9%	95.4%	91.2%	89.9%	88.6%	87.0%	86.2%	83.4%	82.4%	81.5%	80.5%	79.2%	78.8%	78.1%	77.4%	76.6%	76.0%	75.1%	74.4%	73.5%	72.6%
Injection	7	99.7%	99.6%	100.0%	99.1%	97.8%	96.2%	95.4%	94.1%	92.4%	89.6%	86.9%	76.4%	74.5%	71.8%	71.2%	71.2%	70.7%	67.4%	67.3%	64.2%	61.3%	59.5%	58.4%	58.4%

1.Oral 2.Injection

2. Analysis by Attributes

[Product Charateristics] - Route of administration

Graph 6. Sales growth rate of original drugs by formulation, based on volume

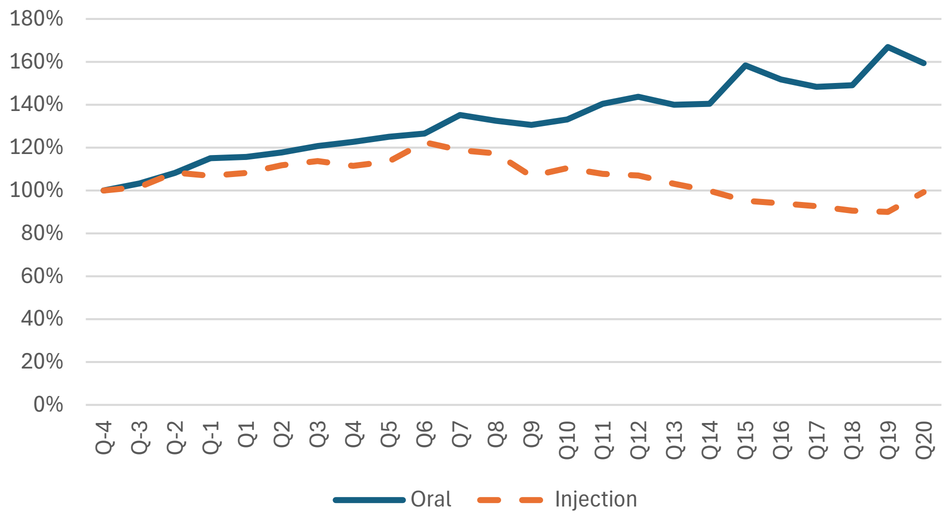

Q1 = the quarter first generic launched

Statistical analysis results

Formulation	N		Estimate	Std. Error	t value	Pr(>|t|)
Oral	41	Reference				
Injection	7		-0.27301	0.04098	-6.663	2.93e-08 ***

Signif. codes: '***' 0.001 '**' 0.01 '*' 0.05 '.' 0.1 '

Formulation	N	Q-4	Q-3	Q-2	Q-1	Q1	Q2	Q3	Q4	Q5	Q6	Q7	Q8	Q9	Q10	Q11	Q12	Q13	Q14	Q15	Q16	Q17	Q18	Q19	Q20
Oral	41	100.0%	103.3%	108.2%	115.0%	115.6%	117.8%	120.8%	122.6%	125.0%	126.6%	135.1%	132.5%	130.6%	133.1%	140.5%	143.8%	140.0%	140.4%	158.3%	151.7%	148.3%	149.1%	166.9%	159.4%
Injection	7	100.0%	101.6%	108.3%	106.8%	108.2%	111.8%	113.8%	111.5%	113.6%	122.5%	118.7%	117.3%	106.7%	110.4%	107.7%	107.0%	103.1%	99.8%	95.1%	94.0%	92.7%	90.6%	90.0%	99.2%

1.Oral 2.Injection

2. Analysis by Attributes [Generic competition] - #of generic drug

Graph 7. Market share of original drugs in the same molecule market by number of generic drugs launched in the 5 years after first generic drug launch, based on value

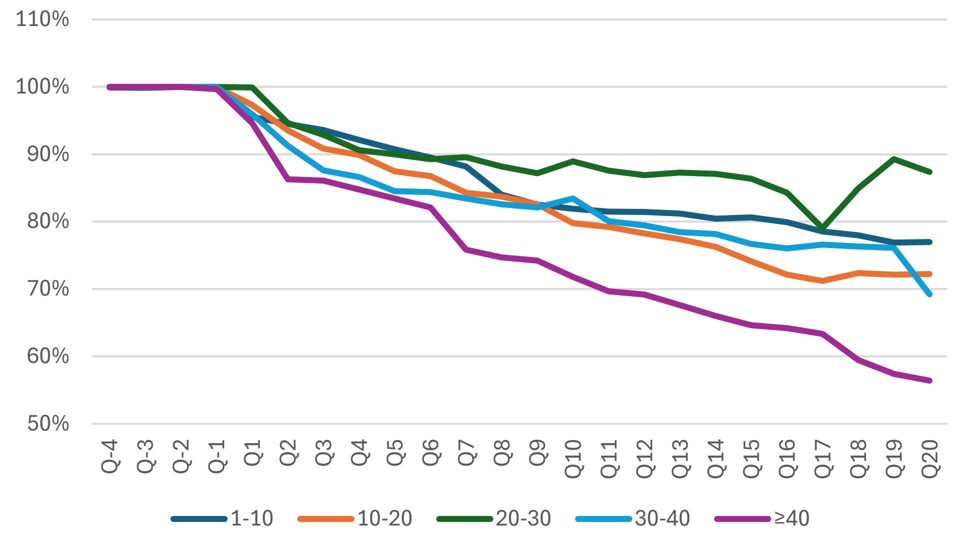

1. More than 1 to less than 10 2. More than 10 to less than 20 3. More than 20 to less than 30 4. More than 30 to less than 40 5. More than 40

Q1 = the quarter first generic launched

Statistical analysis results

#of generic group	N		Estimate	Std. Error	t value	Pr(>|t|)
1-10	22	Reference				
10-20	8		-0.02799	0.02853	-0.981	0.328669
20-30	2		0.03456	0.02853	1.211	0.228196
30-40	3		-0.02460	0.02853	-0.862	0.390313
≥40	13		-0.09858	0.02853	-3.455	0.000771 ***

1. More than 1 to less than 10 2. More than 10 to less than 20 3. More than 20 to less than 30 4. More than 30 to less than 40 5. More than 40
Signif. codes: '***' 0.001 '**' 0.01 '*' 0.05 '.' 0.1

#of generics	N	Q-4	Q-3	Q-2	Q-1	Q1	Q2	Q3	Q4	Q5	Q6	Q7	Q8	Q9	Q10	Q11	Q12	Q13	Q14	Q15	Q16	Q17	Q18	Q19	Q20
1-10	22	99.9%	99.9%	100.0%	99.7%	95.5%	94.5%	93.6%	92.2%	90.8%	89.5%	88.2%	84.0%	82.6%	81.9%	81.5%	81.4%	81.2%	80.4%	80.7%	79.9%	78.6%	78.0%	76.9%	77.0%
10-20	8	100.0%	100.0%	100.0%	100.0%	97.3%	93.6%	90.8%	89.9%	87.5%	86.8%	84.3%	83.7%	82.6%	79.8%	79.2%	78.3%	77.4%	76.3%	74.1%	72.1%	71.2%	72.4%	72.1%	72.2%
20-30	2	100.0%	100.0%	100.0%	100.0%	99.9%	94.7%	92.9%	90.6%	90.0%	89.3%	89.6%	88.2%	87.2%	89.0%	87.6%	86.9%	87.3%	87.1%	86.4%	84.3%	79.0%	85.0%	89.3%	87.4%
30-40	3	100.0%	100.0%	100.0%	100.0%	95.9%	91.3%	87.6%	86.6%	84.5%	84.4%	83.5%	82.6%	82.1%	83.4%	80.1%	79.4%	78.4%	78.1%	76.7%	76.0%	76.6%	76.3%	76.1%	69.2%
≥40	13	100.0%	100.0%	100.0%	99.7%	94.6%	86.3%	86.1%	84.8%	83.4%	82.1%	75.8%	74.7%	74.2%	71.8%	69.7%	69.2%	67.6%	66.0%	64.6%	64.2%	63.3%	59.4%	57.4%	56.4%

1. More than 1 to less than 10 2. More than 10 to less than 20 3. More than 20 to less than 30 4. More than 30 to less than 40 5. More than 40

2. Analysis by Attributes [Generic competition] - #of generic drug

Graph 8. Sales growth rate of original drugs by number of generic drugs launched in the 5 years after first generic drug launch , based on volume

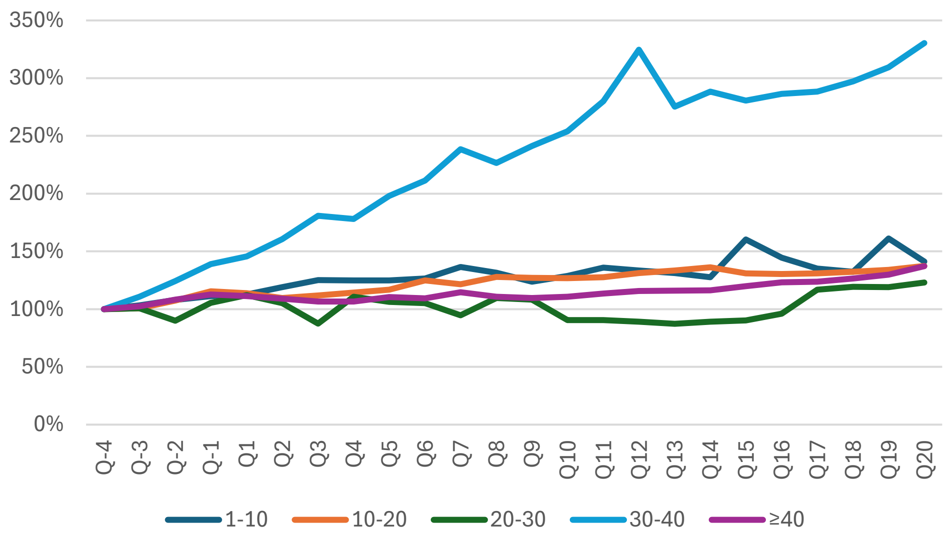

1. More than 1 to less than 10 2. More than 10 to less than 20 3. More than 20 to less than 30 4. More than 30 to less than 40 5. More than 40

Q1 = the quarter first generic launched

Statistical analysis results

#of generic group	N		Estimate	Std. Error	t value	Pr(>|t|)
1-10	22	Reference				
10-20	8		-0.05644	0.09683	-0.583	0.56109
20-30	2		-0.26316	0.09683	-2.718	0.00759 **
30-40	3		0.99621	0.09683	10.288	< 2e-16 ***
≥40	13		-0.13927	0.09683	-1.438	0.15307

1. More than 1 to less than 10 2. More than 10 to less than 20 3. More than 20 to less than 30 4. More than 30 to less than 40 5. More than 40
Signif. codes: '***' 0.001 '**' 0.01 '*' 0.05 '.' 0.1

#of generics	N	Q-4	Q-3	Q-2	Q-1	Q1	Q2	Q3	Q4	Q5	Q6	Q7	Q8	Q9	Q10	Q11	Q12	Q13	Q14	Q15	Q16	Q17	Q18	Q19	Q20
1-10	22	100.0%	103.3%	107.9%	111.3%	112.6%	119.2%	125.1%	125.0%	124.8%	126.4%	136.5%	131.5%	123.6%	128.8%	136.1%	133.4%	131.2%	127.8%	160.2%	144.5%	135.2%	132.5%	161.2%	141.3%
10-20	8	100.0%	100.8%	107.3%	115.5%	113.9%	109.7%	111.8%	114.2%	116.8%	124.9%	121.6%	128.0%	127.1%	126.8%	127.7%	131.3%	133.5%	136.2%	131.0%	130.5%	130.9%	132.4%	133.9%	137.4%
20-30	2	100.0%	100.7%	89.9%	105.4%	112.2%	105.2%	87.4%	110.6%	106.3%	105.3%	94.7%	109.7%	108.3%	90.6%	90.4%	89.2%	87.3%	89.1%	90.4%	96.1%	116.9%	119.3%	119.0%	123.0%
30-40	3	100.0%	110.7%	124.4%	139.1%	145.7%	160.5%	180.8%	178.2%	197.9%	211.4%	238.5%	226.5%	241.3%	253.9%	280.2%	324.6%	275.2%	288.3%	280.5%	286.6%	288.4%	297.1%	309.5%	330.4%
≥40	13	100.0%	102.4%	108.3%	112.6%	111.4%	109.1%	106.5%	106.7%	110.4%	109.4%	114.7%	110.8%	109.7%	110.8%	113.6%	115.7%	116.0%	116.2%	120.0%	123.2%	123.6%	126.6%	129.9%	137.2%

1. More than 1 to less than 10 2. More than 10 to less than 20 3. More than 20 to less than 30 4. More than 30 to less than 40 5. More than 40

2. Analysis by Attributes [Market attractiveness] - Product sales before the first generic drugs launch

Graph 9. Market share of Original drugs in the same molecule market by original drug's sales before first generic drug launched, based on value

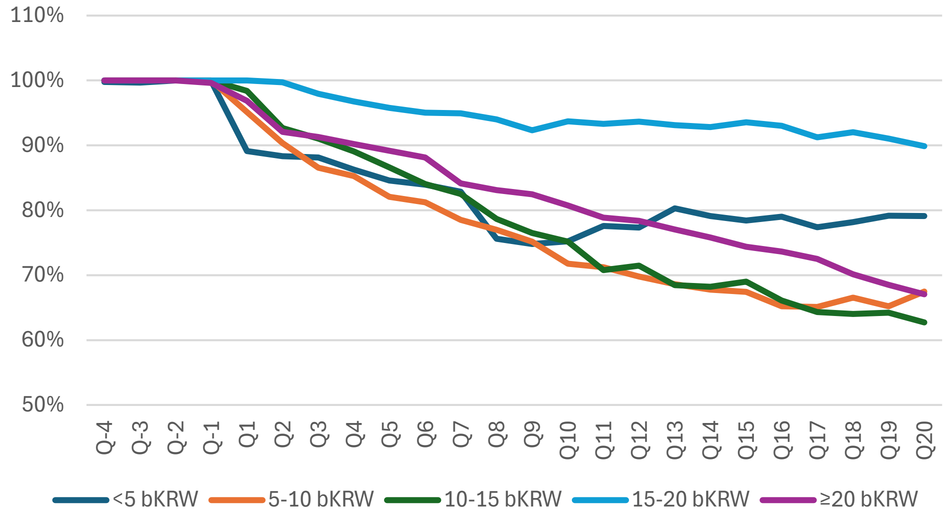

1. Less than 5 billion won 2. More than 5 billion won but less than 10 billion won 3. More than 10 billion won but less than 15 billion won 4. More than 15 billion won but less than 20 billion won 5. More than 20 billion won, Local currency

Q1 = the quarter first generic launched

Statistical analysis results

Product sales before first generic drug launch	N		Estimate	Std. Error	t value	Pr(>|t|)
<5 bKRW	8	Reference				
5-10 bKRW	6		-4.870e-02	2.994e-02	-1.626	0.10659
10-15 bKRW	5		-3.736e-02	2.994e-02	-1.248	0.21459
15-20 bKRW	4		1.132e-01	2.994e-02	3.780	0.00025 ***
≥20 bKRW	25		-1.458e-05	2.994e-02	0.000	0.99961

1. Less than 5 billion won 2. More than 5 billion won but less than 10 billion won 3. More than 10 billion won but less than 15 billi on won 4. More than 15 billion won but less than 20 billion won 5. More than 20 billion won, Local currency
Signif. codes: '***' 0.001 '**' 0.01 '*' 0.05 '.' 0.1

	N	Q-4	Q-3	Q-2	Q-1	Q1	Q2	Q3	Q4	Q5	Q6	Q7	Q8	Q9	Q10	Q11	Q12	Q13	Q14	Q15	Q16	Q17	Q18	Q19	Q20
<5 bKRW	8	99.8%	99.7%	100.0%	99.9%	89.1%	88.3%	88.2%	86.3%	84.6%	83.9%	82.8%	75.6%	74.8%	75.2%	77.6%	77.3%	80.3%	79.1%	78.4%	79.0%	77.4%	78.2%	79.2%	79.1%
5-10 bKRW	6	100.0%	100.0%	100.0%	100.0%	95.1%	90.3%	86.6%	85.3%	82.1%	81.2%	78.5%	77.0%	75.2%	71.8%	71.2%	69.8%	68.6%	67.8%	67.4%	65.2%	65.1%	66.5%	65.2%	67.5%
10-15 bKRW	5	100.0%	100.0%	100.0%	100.0%	98.4%	92.7%	91.0%	89.1%	86.6%	84.1%	82.5%	78.7%	76.5%	75.2%	70.8%	71.5%	68.5%	68.2%	69.0%	66.1%	64.3%	64.0%	64.2%	62.7%
15-20 bKRW	4	100.0%	100.0%	100.0%	100.0%	100.0%	99.7%	98.0%	96.8%	95.8%	95.1%	94.9%	94.0%	92.3%	93.7%	93.3%	93.6%	93.1%	92.8%	93.5%	93.0%	91.3%	92.0%	91.1%	89.9%
≥20 bKRW	25	100.0%	100.0%	100.0%	99.6%	96.9%	92.1%	91.3%	90.2%	89.2%	88.1%	84.1%	83.1%	82.4%	80.8%	78.9%	78.4%	77.1%	75.8%	74.4%	73.6%	72.5%	70.1%	68.5%	67.1%

1. Less than 5 billion won 2. More than 5 billion won but less than 10 billion won 3. More than 10 billion won but less than 15 billion won 4. More than 15 billion won but less than 20 billion won 5. More than 20 billion won

2. Analysis by Attributes [Market attractiveness] - Product sales before the first generic drugs launch

Graph 10. Sales growth rate of original drugs by original drug's sales before first generic drug launched, based on volume

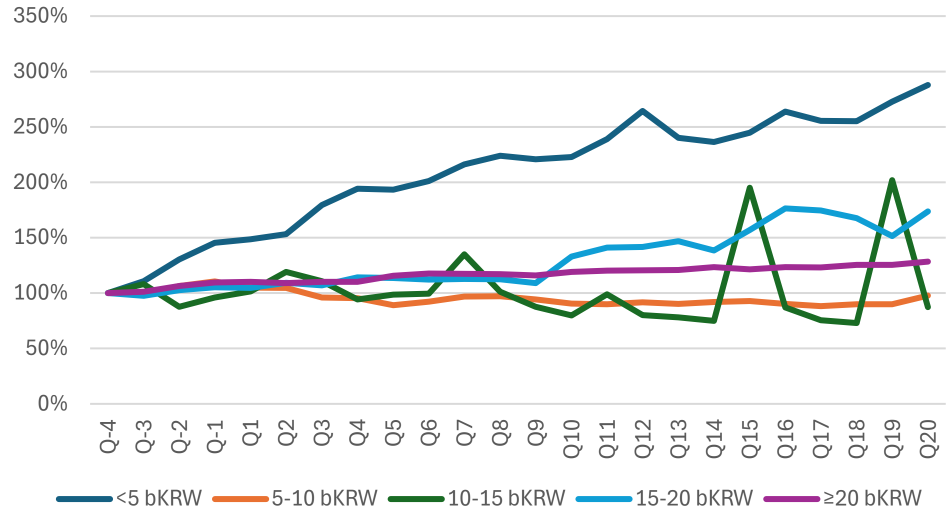

1. Less than 5 billion won 2. More than 5 billion won but less than 10 billion won 3. More than 10 billion won but less than 15 billion won 4. More than 15 billion won but less than 20 billion won 5. More than 20 billion won, Local currency

Q1 = the quarter first generic launched

Statistical analysis results

Product sales before first generic drug launch	N		Estimate	Std. Error	t value	Pr(>|t|)
<5 bKRW	8	Reference				
5-10 bKRW	6		-1.12894	0.08861	-12.741	< 2e-16 ***
10-15 bKRW	5		-1.05359	0.08861	-11.890	< 2e-16 ***
15-20 bKRW	4		-0.79099	0.08861	-8.927	8.18e-15 ***
≥20 bKRW	25		-0.92019	0.08861	-10.385	< 2e-16 ***

1. Less than 5 billion won 2. More than 5 billion won but less than 10 billion won 3. More than 10 billion won but less than 15 billion won 4. More than 15 billion won but less than 20 billion won 5. More than 20 billion won, Local currency
Signif. codes: '***' 0.001 '**' 0.01 '*' 0.05 '.' 0.1

	N	Q-4	Q-3	Q-2	Q-1	Q1	Q2	Q3	Q4	Q5	Q6	Q7	Q8	Q9	Q10	Q11	Q12	Q13	Q14	Q15	Q16	Q17	Q18	Q19	Q20
<5 bKRW	8	100.0%	110.6%	130.5%	145.3%	148.6%	153.3%	179.5%	194.3%	193.3%	201.0%	216.1%	224.0%	220.8%	222.7%	239.0%	264.4%	240.2%	236.3%	244.8%	263.9%	255.5%	255.2%	272.8%	287.8%
5-10 bKRW	6	100.0%	99.4%	106.4%	110.5%	105.0%	104.8%	95.9%	95.3%	89.2%	92.3%	96.8%	97.2%	94.4%	90.5%	90.0%	91.6%	90.2%	92.0%	92.8%	90.2%	88.2%	89.9%	89.9%	97.8%
10-15 bKRW	5	100.0%	108.1%	87.7%	96.1%	101.5%	119.1%	110.6%	94.2%	98.7%	99.5%	135.0%	101.1%	87.5%	79.7%	98.8%	80.1%	78.1%	75.0%	194.9%	87.1%	75.5%	72.9%	201.9%	87.2%
15-20 bKRW	4	100.0%	97.5%	102.6%	105.2%	104.8%	109.2%	106.9%	114.1%	113.7%	112.1%	112.9%	112.4%	109.1%	133.0%	141.1%	141.7%	146.8%	138.5%	156.9%	176.4%	174.5%	167.6%	151.6%	173.6%
≥20 bKRW	25	100.0%	101.3%	106.5%	109.5%	110.1%	108.9%	110.3%	110.1%	115.7%	117.6%	117.4%	117.0%	115.9%	119.1%	120.1%	120.5%	120.9%	123.3%	121.5%	123.4%	123.2%	125.3%	125.4%	128.4%

1. Less than 5 billion won 2. More than 5 billion won but less than 10 billion won 3. More than 10 billion won but less than 15 billion won 4. More than 15 billion won but less than 20 billion won 5. More than 20 billion won

2. Analysis by Attributes [Market attractiveness] - Market size before first generic drugs launch

Graph 11. Market share of original drugs in the same molecule market by Therapeutic market size (ATC 4 Level) before first generic launched, based on value

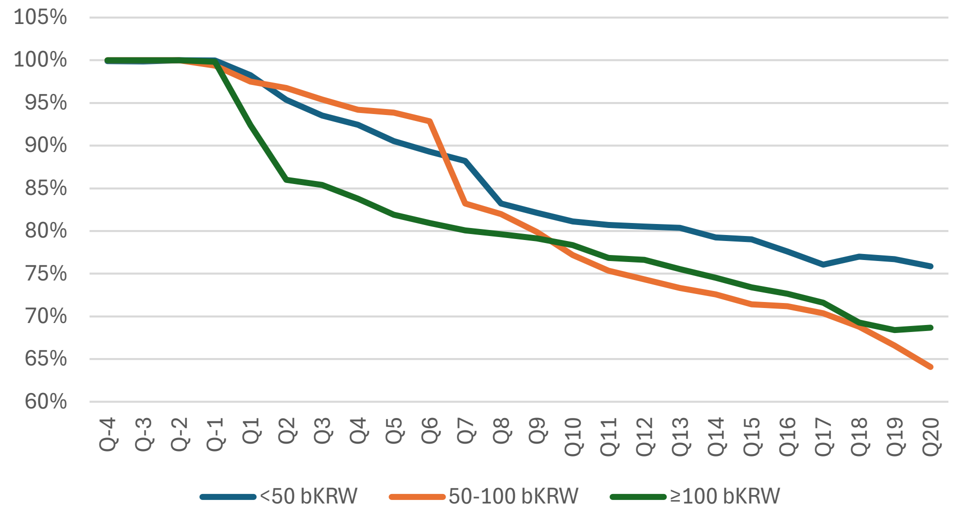

1. Less than 50 billion won 2. More than 50 billion won ~ less than 100 billion won 3. More than 100 billion won, Local currency

Q1 = the quarter first generic launched

Statistical analysis results

Market sales before first generic launch	N		Estimate	Std. Error	t value	Pr(>|t|)
<50 bKRW	19	Reference				
50-100 bKRW	10		-0.03223	0.03087	-1.044	0.300
≥100 bKRW	19		-0.05083	0.03087	-1.647	0.104

1. Less than 50 billion won 2. More than 50 billion won ~ less than 100 billion won 3. More than 100 billion won
Signif. codes: '***' 0.001 '**' 0.01 '*' 0.05 '.' 0.1

	N	Q-4	Q-3	Q-2	Q-1	Q1	Q2	Q3	Q4	Q5	Q6	Q7	Q8	Q9	Q10	Q11	Q12	Q13	Q14	Q15	Q16	Q17	Q18	Q19	Q20
<50 bKRW	19	99.9%	99.9%	100.0%	100.0%	98.3%	95.4%	93.5%	92.4%	90.5%	89.3%	88.2%	83.2%	82.1%	81.1%	80.7%	80.5%	80.4%	79.2%	79.0%	77.6%	76.1%	77.0%	76.7%	75.9%
50-100 bKRW	10	100.0%	100.0%	100.0%	99.4%	97.5%	96.7%	95.4%	94.2%	93.8%	92.8%	83.2%	82.0%	79.9%	77.2%	75.3%	74.4%	73.3%	72.6%	71.4%	71.2%	70.3%	68.8%	66.6%	64.1%
≥100 bKRW	19	100.0%	100.0%	100.0%	99.8%	92.4%	86.0%	85.4%	83.8%	81.9%	80.9%	80.1%	79.6%	79.1%	78.4%	76.9%	76.6%	75.5%	74.5%	73.4%	72.7%	71.6%	69.3%	68.4%	68.7%

1. Less than 50 billion won 2. More than 50 billion won ~ less than 100 billion won 3. More than 100 billion won

2. Analysis by Attributes [Market attractiveness] - Market size before first generic drugs launch

Graph 12. Sales growth rate of original drugs by Therapeutic market size (ATC 4 Level) before first generic launched, based on volume

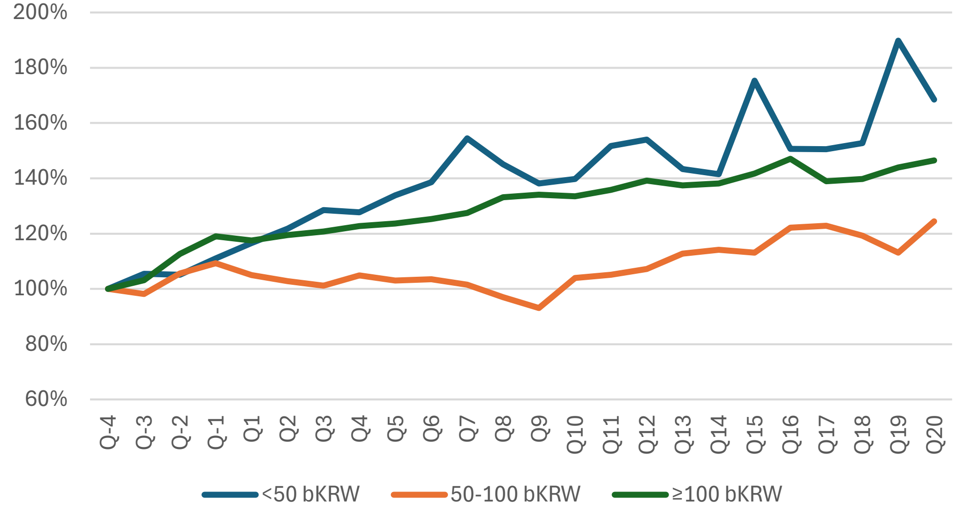

1. Less than 50 billion won 2. More than 50 billion won ~ less than 100 billion won 3. More than 100 billion won, Local currency

Q1 = the quarter first generic launched

Statistical analysis results

Market sales before first generic launch	N		Estimate	Std. Error	t value	Pr(>|t|)
<50 bKRW	19	Reference				
50-100 bKRW	10		-0.31740	0.04535	-6.999	1.34e-09 ***
≥100 bKRW	19		-0.10132	0.04535	-2.234	0.0287 *

1. Less than 50 billion won 2. More than 50 billion won ~ less than 100 billion won 3. More than 100 billion won
Signif. codes: '***' 0.001 '**' 0.01 '*' 0.05 '.' 0.1 '

	N	Q-4	Q-3	Q-2	Q-1	Q1	Q2	Q3	Q4	Q5	Q6	Q7	Q8	Q9	Q10	Q11	Q12	Q13	Q14	Q15	Q16	Q17	Q18	Q19	Q20
<50 bKRW	19	100.0%	105.4%	105.1%	111.1%	116.6%	121.8%	128.5%	127.7%	133.8%	138.6%	154.5%	145.0%	138.1%	139.7%	151.7%	154.0%	143.3%	141.5%	175.3%	150.7%	150.5%	152.7%	189.8%	168.5%
50-100 bKRW	10	100.0%	98.1%	105.5%	109.2%	105.0%	102.8%	101.2%	104.9%	103.1%	103.5%	101.5%	96.9%	93.1%	104.0%	105.1%	107.2%	112.7%	114.1%	113.1%	122.1%	122.9%	119.2%	113.1%	124.4%
≥100 bKRW	19	100.0%	103.2%	112.6%	119.0%	117.5%	119.4%	120.8%	122.7%	123.6%	125.3%	127.4%	133.2%	134.1%	133.5%	135.8%	139.2%	137.4%	138.1%	141.7%	147.1%	139.0%	139.8%	144.0%	146.4%

1. Less than 50 billion won 2. More than 50 billion won ~ less than 100 billion won 3. More than 100 billion won
